# Predicting the mutation effects of protein–ligand interactions via end-point binding free energy calculations: strategies and analyses

**DOI:** 10.1186/s13321-022-00639-y

**Published:** 2022-08-20

**Authors:** Yang Yu, Zhe Wang, Lingling Wang, Sheng Tian, Tingjun Hou, Huiyong Sun

**Affiliations:** 1 Department of Medicinal Chemistry, China Pharmaceutical University, Nanjing, 210009 Jiangsu People’s Republic of China; 2 Innovation Institute for Artificial Intelligence in Medicine of Zhejiang University, College of Pharmaceutical Sciences, Zhejiang University, Hangzhou, 310058 Zhejiang People’s Republic of China; 3 Department of Medicinal Chemistry, College of Pharmaceutical Sciences, Soochow University, Suzhou, 215123 People’s Republic of China

## Abstract

Protein mutations occur frequently in biological systems, which may impact, for example, the binding of drugs to their targets through impairing the critical H-bonds, changing the hydrophobic interactions, etc. Thus, accurately predicting the effects of mutations on biological systems is of great interests to various fields. Unfortunately, it is still unavailable to conduct large-scale wet-lab mutation experiments because of the unaffordable experimental time and financial costs. Alternatively, in silico computation can serve as a pioneer to guide the experiments. In fact, numerous pioneering works have been conducted from computationally cheaper machine-learning (ML) methods to the more expensive alchemical methods with the purpose to accurately predict the mutation effects. However, these methods usually either cannot result in a physically understandable model (ML-based methods) or work with huge computational resources (alchemical methods). Thus, compromised methods with good physical characteristics and high computational efficiency are expected. Therefore, here, we conducted a comprehensive investigation on the mutation issues of biological systems with the famous end-point binding free energy calculation methods represented by MM/GBSA and MM/PBSA. Different computational strategies considering different length of MD simulations, different value of dielectric constants and whether to incorporate entropy effects to the predicted total binding affinities were investigated to provide a more accurate way for predicting the energetic change upon protein mutations. Overall, our result shows that a relatively long MD simulation (e.g. 100 ns) benefits the prediction accuracy for both MM/GBSA and MM/PBSA (with the best Pearson correlation coefficient between the predicted ∆∆*G* and the experimental data of ~ 0.44 for a challenging dataset). Further analyses shows that systems involving large perturbations (e.g. multiple mutations and large number of atoms change in the mutation site) are much easier to be accurately predicted since the algorithm works more sensitively to the large change of the systems. Besides, system-specific investigation reveals that conformational adjustment is needed to refine the micro-environment of the manually mutated systems and thus lead one to understand why longer MD simulation is necessary to improve the predicting result. The proposed strategy is expected to be applied in large-scale mutation effects investigation with interpretation.

**Graphical Abstract:**

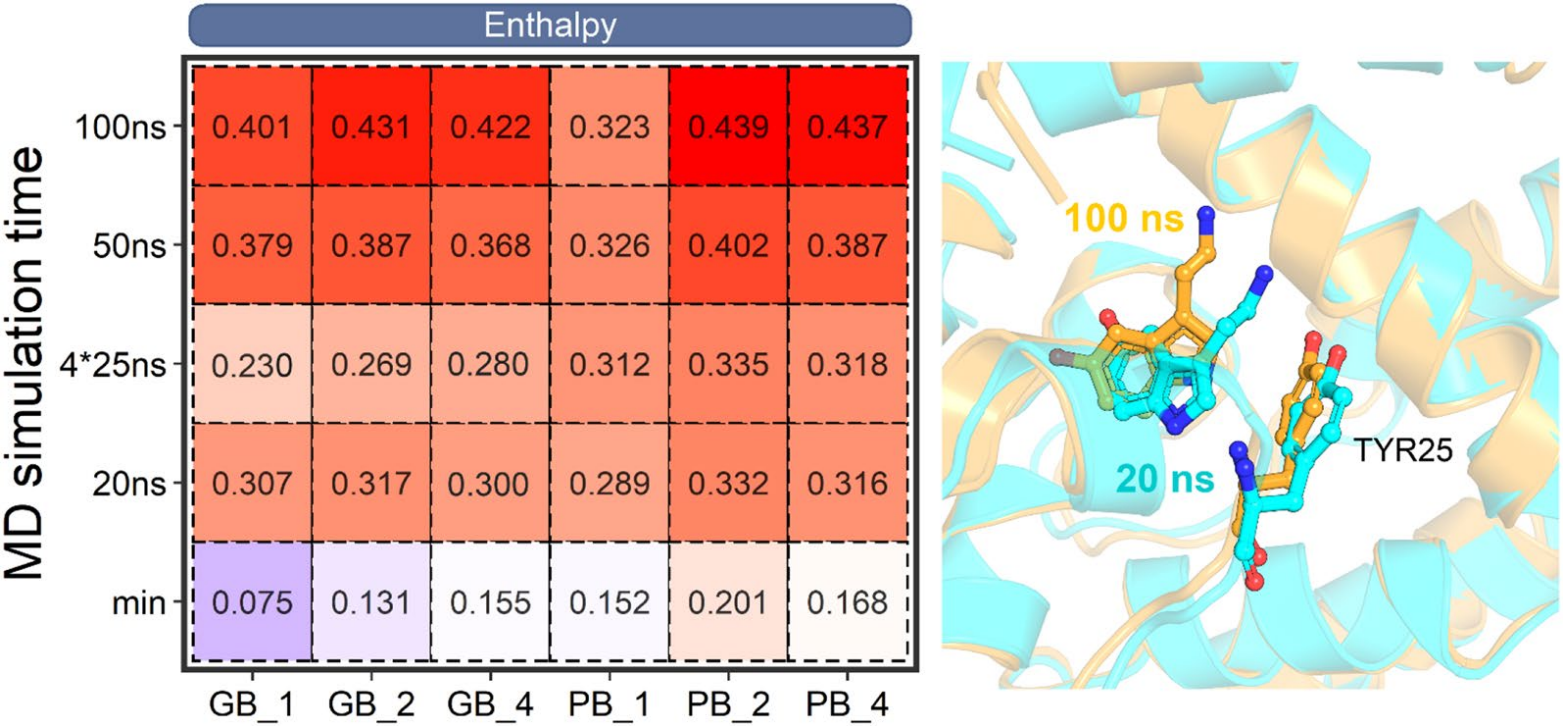

**Supplementary Information:**

The online version contains supplementary material available at 10.1186/s13321-022-00639-y.

## Introduction

Chemical thermodynamics and kinetics play foundational roles in regulating biological processes [1–4], such as the processes of protein–protein interactions [5], protein-metabolite recognitions [6] and protein-nucleic acids interactions [7, 8], where binding free energy between the systems controls the tendency of the spontaneous reactions. However, these spontaneous reactions may be broken by perturbations occurring in the systems, such as mutations in the protein, which may seriously affect the binding affinities of i.e. drugs to their targets (namely drug resistance), protein–protein interactions (PPI), through impairing the critical H-bonds and/or changing the hydrophobic interactions. Thus, accurately predicting the binding energy change of the perturbed (mutated) systems has always been an essential question in various biological issues. Unfortunately, it is still unavailable to conduct large-scale wet-lab mutation experiments because of the unaffordable experimental time and financial costs. Alternatively, with the development of the computational hardware (i.e. GPU acceleration) and advanced algorithms (i.e. artificial intelligence) in recent years, in silico computation can serve as a pioneer to guide the experiments.

Actually, numerous pioneering theoretical works have been conducted on the mutated systems to predict the change of the free energy for issues such as drug resistance [9–11], protein–protein interactions [12, 13]. Generally, the predicting approaches can be divided into two categories, namely the statistics-based methods using machine learning (ML) and the structure-based methods using physical models (such as those applying force fields). Although the ML-based methods usually exhibit higher computational efficiency and accuracy compared with the physics-based approaches [14, 15], these methods may usually suffer from the problems of difficulty for mechanism explanation. And the ML-based methods tend to show a limited scope of application due to the biased or limited training set. On the other hand, structure-based methods such as force field guided free energy calculations exhibit more advantages in the issues of model generalizability and interpretability, and can be used in various types of predictions due to the shared physical foundation [13, 16–19]. In these algorithms, alchemical methods represented by free energy perturbation (FEP) and thermodynamics integration (TI) may be the most theoretically rigorous and precise ones with an average error of ~ 1 kcal/mol against the experimental data (i.e. *FEP*+ [20]). However, these approaches may be fall into the other extreme of requiring huge computational resources and convergence issues, and are usually hard to be applied in large-scale drug design campaigns.

Nevertheless, between these two extremes (ML-based models and alchemical methods), there also exist compromised methods that are able to provide not only reasonable accuracy with good generalizability, but also relatively fast computational efficiency with great interpretability. End-point binding free energy calculation methods represented by MM/GB(PB)SA (molecular mechanics [MM] with generalized Born [GB]/Poisson-Boltzmann [PB] and surface area [SA]) [21] are one of the most famous methods in this area, which calculate the binding free energy of the systems using only the initial (i.e. conformations of the receptor and ligand) and the final states (conformation of the complex), the so-called two-end-state methods. The physics-based algorithm offers a more reliable physical reality than ML-based approaches and exhibits a much faster computational efficiency compared with the alchemical methods, thus making these methods successfully used in various situations, such as drug resistance/selectivity mechanism analyses [22, 23].

Numerous mutation associated studies have been conducted with the end-point binding free energy calculation methods for various purposes, such as understanding the drug resistance mechanisms for specific targets [19, 23–28], identifying hot-spot residues responsible for protein–protein interactions [13, 29–32], or protein stabilities [33]. For instance, Ikemura et al. have conducted MM/GBSA and MM/PBSA calculations to predict the drug sensitivity of several EGFR inhibitors upon rare mutations [24], and they successfully predicted the diverse sensitivities of exon 20 insertion mutants with very high correlation against the experimental data (*r*^2^ = 0.72, n = 9). Using MD simulation and MM/PBSA calculation, Fulle et al. revealed that the drug resistance of linezolid in the large ribosomal subunit is caused by the long-range propagated mutation effects (with the drug-mutation distance > 10 Å) [25], demonstrating that MD simulation is capable of capturing long-range mutation effects. Zhang et al. have investigated the dynamic effects of the mutations in the SARS-CoV-2 spike protein with MD simulation and MM/PBSA calculation, and they demonstrated that several mutations on the spike protein (V367F and N354D/D364Y) may enhance its binding to hACE2. Moreover, Schrodinger Inc. has conducted two comprehensive analyses with FEP and end-point binding free energy calculations on massive mutations for PPI hot-spot identification [13], and protein-stability investigation [33], and reasonable accuracies are shown of the two studies (*r*_p_ = 0.45 ~ 0.6). Although significant correlations are usually shown of these works against the experimental data, most of the studies have been conducted on individual systems and, up to now, there is no comprehensive study to propose a general rule for accurately predicting the mutation effects on protein–ligand systems. Therefore, in this study, we have systematically assessed the performance of MM/GBSA and MM/PBSA approaches on the mutated systems with the consideration of different MD simulation times, dielectric constants and entropy effects. A dataset containing 89 single/multiple mutations within 13 diverse proteins and 20 ligands was used for this study (Table S1 in Additional file 1). The result shows that the MD simulation time can significantly affect the performance of the calculated binding free energies for both MM/GBSA and MM/PBSA. Further investigation shows that systems suffering from large perturbations (e.g. multiple mutations or large number of atoms change in the mutation site) are much easier to be accurately predicted because the algorithm usually works sensitively to the large change of the systems. Moreover, to understand the detailed mechanism of the MD simulation time on the mutated systems, a representative system was used to intuitively reveal how the MD simulation time explicitly affects the prediction result.

## Methods

### Dataset preparation

Consisting of 13 proteins and 20 ligands, a dataset containing 89 single/multiple mutations from Aldeghi’s work (Platinum database) was used for this study [34, 35]. It should be noted that ligands containing the phosphate group were excluded from the original dataset since unreasonable large atomic charges were usually assigned by the atomic charge fitting algorithm. Besides, one more thing needs to be noted that a part of the mutation sites were wrongly recorded in the supporting material of the original database (the amino acid of the wild-type and the mutants were reversely recorded), thus we have corrected these mutations and the full mutations were listed in Additional file 1: Table S1.

In the preparation of the systems, all the mutants without a crystal structure were manually mutated from the corresponding protein–ligand structure. In detail, the mutations were introduced with the “*Build and Edit Protein*” module in Discovery Studio 2019 (Accelrys Inc., http://www.accelrys.com), followed by the geometry refinement and CHARMm force field optimization for the purpose of cleaning the structural bumps between the manually introduced mutations and the surroundings.

The resulted systems were prepared with *antechamber* and *tleap* modules in AMBER/20 simulation package [36, 37]. Considering the comparable performance in the binding free energy calculation and much lower computational cost compared with the RESP charges [38], AM1-BCC atomic charges [39] were employed for all ligands for the subsequent calculations. The small molecules were parameterized with the general amber force field (*gaff*, version 1.81) [40], while amber ff14SB force field [41] was used for the simulation of the proteins. To balance the redundant charges, counterions of Na^+^ and Cl^−^ were added to the ligand–protein systems. Each ligand–protein complex was immersed in a cubic TIP3P water box with a 10 Å boundary [42].

### Molecular mechanics (MM) minimization

For MM minimization, the real-space cutoff distance (including the van der Waals and short-range electrostatic interactions) was set to 10 Å, while the PME algorithm (particle mesh Ewald) [43] was used to treat the long-range electrostatic interactions [44]. All the systems were optimized with four steps of minimizations. Note that the manually introduced mutations may impact the conformation of the surrounding residues. Here, different optimization strategies were conducted for the full-crystal systems and the in silico-mutation-containing systems in the first step of the minimization, in which all the hydrogen atoms were released with others atoms constrained for the full-crystal systems, whereas all the hydrogen atoms and the heavy atoms within 5 Å of the mutations (including the heavy atoms in amino acids, ligand, solvent and the mutation itself) were optimized with other atoms constrained for the in silico-mutation-containing systems; next, heavy atom (oxygen atom) in water and counterions (Na+/Cl−) were released; then, the sidechains in residues were additionally set free for optimization; and finally, all the atoms were released for full minimization. In each step, 5000 steps of minimization, including 1000 circles of steepest descent and 4000 cycles of conjugate gradient minimizations, were conducted with an elastic constant of 5 kcal/mol Å^2^ of the constraint on the systems.

### Molecular dynamics (MD) simulation

In the process of MD simulation, three steps of MD simulation were conducted for each system. First, the temperature of the system was heated from 0 to 300 K during a period of 50 ps in the NVT ensemble, in which heavy atoms in the protein backbone were constrained with an elastic constant of 2 kcal/mol∙Å^2^. Then, a 50 ps of density equilibration was carried out in the NTP ensemble (*T* = 300 K and *P* = 1 atm) with the same constraint on the heavy atoms of the protein backbone as that of the heating process. Finally, a 100 ns MD simulation was performed in the NPT ensemble without any restraint. In all the MD simulations, the time step was set to 2 fs with the SHAKE algorithm [45] constraining the covalent bonds between the hydrogen atoms and the connected heavy atoms. The coordinates (the MD trajectory) were collected with an interval of 5 ps (25,000 steps), thus a total of 2000 frames were collected for each system.

Moreover, to fully investigate the sampling effect of MD based on one single long trajectory (100 ns), four short MD simulations (25 ns) were additionally conducted using random seeds with the same parameters/process as that of the 100 ns MD simulation for each system. All the MD simulations were performed with the *pmemd.cuda* module in AMBER/20.

### End-point binding free energy calculations with MM/GBSA and MM/PBSA

The four 25 ns and one 100 ns MD trajectories of each system were used for the MM/GBSA and MM/PBSA calculations. In the MM/PBSA and MM/GBSA approaches, the free energy for binding of a ligand to the receptor (Eq. 1) can be decomposed into different energetic contributions as expressed below [46]:

1 $$\Delta {G}_{bind}={G}_{complex}-\left({G}_{receptor}-{G}_{ligand}\right)$$

2 $${\Delta G}_{bind}={\Delta E}_{MM}+{\Delta G}_{sol}-T\Delta S$$

3 $$\Delta {E}_{MM}={\Delta E}_{bond}+{\Delta E}_{angle}+{\Delta E}_{dihedral}{+{\Delta E}_{ele}+\Delta E}_{vdW}$$

4 $$\Delta {G}_{sol}={\Delta G}_{PB/GB}+{\Delta G}_{SA}$$

5 $$\Delta {G}_{SA}=\gamma *SASA+b$$

where Δ*E*_MM_, Δ*G*_sol_, and -TΔ*S* represent the energy contributions of the molecular mechanics energy, the solvation free energy and the entropy upon ligand binding, respectively (Eq. 2), in which Δ*E*_MM_ consists of five energetic terms, namely the bond (Δ*E*_bond_), the angle (Δ*E*_angle_), the dihedral (Δ*E*_dihedral_), the electrostatic (Δ*E*_ele_) and the van der Waals (Δ*E*_vdW_) energies (Eq. 3). Here, we applied the single MD trajectory protocol for the end-point binding free energy calculation for the reasons to derive stable results. Therefore, Δ*E*_bond_, Δ*E*_angle_ and Δ*E*_dihedral_ can be well canceled out in the following calculations, leaving Δ*E*_MM_ being the sum of Δ*E*_vdW_ and Δ*E*_ele_. Δ*G*_sol_ is composed of the polar (Δ*G*_PB/GB_) and the nonpolar (Δ*G*_SA_) contributions to the solvation energy (Eq. 4), where the polar part of the solvation energy can be calculated by either GB or PB model and the nonpolar part of the solvation energy can be calculated with the solvent accessible surface area (SASA) using LCPO algorithm (Eq. 5) [47]. Here, the modified GB model developed by Onufriev (GB^OBC1^) [48] and the PB model parameterized by Tan and Luo (PB^pbsa^) [49] were employed for the polar solvation energy calculations (Δ*G*_PB/GB_) [38, 50]. Since the interior dielectric constant (*ε*_in_) can significantly affect the electrostatic parts (Δ*E*_ele_ and Δ*G*_PB/GB_) of the resulted binding free energy as shown in previous studies [51, 52], herein we tested *ε*_in_ taking 1, 2 and 4 for a comprehensive comparison. The outer dielectric constant was set to 80 to mimic the high dielectric effect of the water environment. The parameters of *γ* and *b* were set to 0.0072 and 0, respectively, for the calculation of Δ*G*_SA_ (Eq. 5). All the MM/GBSA and MM/PBSA calculations were performed with the *MMPBSA.py* module [53] in AMBER simulation package.

### Conformational entropy estimated by NMA

Normal mode analysis (NMA) was used to estimate the conformational entropy of the system upon ligand binding (termed as normal mode entropy, NME). To save the computational cost, the structure-truncation strategy [54] was employed to speed up the NME calculations, where a cutoff of 9 Å was set to truncate the protein around the ligand as the reasonable performance in our previous work [52]. In the truncation of the protein structure, if any heavy atoms of a residue drop into the cutoff sphere, the whole residue is taken into the truncated structure. All the discontinuous residues were treated with charged terminals (COO^−^ and NH_3_^+^) as the better performance in our previous work [52]. For each system, 50 and 12 frames were collected from the 100 ns and each 25 ns MD trajectories, respectively, for the entropy calculation (with equal interval of 2 ns/frame). The maximum optimizing step was set to 10,000, and the convergence condition was set to 1 × 10^–4^. All the NME calculations were conducted by the nmode module in *MMPBSA.py* [53].

### Analysis

In this study, the binding free energy difference between the mutated and the wild-type systems was reported (∆∆*G* = ∆*G*_MT_-∆*G*_WT_). Pearson correlation coefficient was employed to compare the results resulted from different computational strategies, and the standard deviation of the Pearson correlation coefficient was estimated by randomly selecting 80% data for 100 times. All the results can be found in GitHub (https://github.com/yuyangniuer/MUTATION).

### Hardware and computational cost

We performed MD simulations and MM/PB(GB)SA calculations on a 384 GB-memory Linux Cluster (Centos7 operating system) with 12 NVIDIA GeForce RTX 2080Ti graphics cards and Intel Xeon Gold 5120 processors (2.2 GHz, a total of 168 CPU cores). Typically, for a system containing ~ 40,000 atoms (~ 300 residues), the computational cost for a 100 ns MD simulation and 2000 frames of MM/PB(GB)SA calculation is about 8 ~ 10 h on one GPU card and 14 CPU cores.

## Results and discussion

### Overall features of the investigated systems

**Fig. 1:**
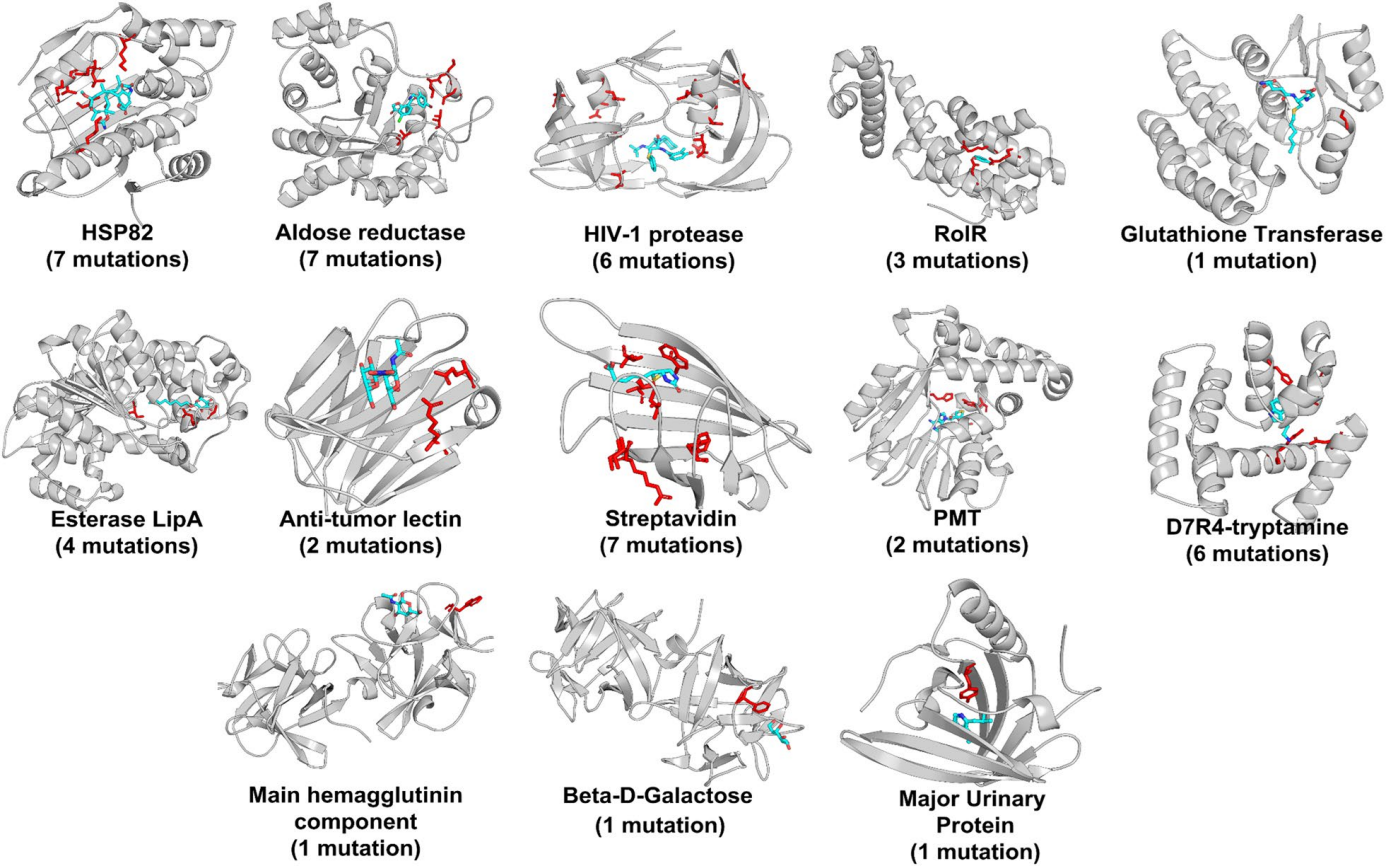
Structural illustration of the 13 investigated targets and the corresponding mutation sites (red) and ligands (cyan)

Herein, 13 targets belonging to different protein families with 20 small molecular ligands were employed for the investigation (Fig. 1), which construct a total of 89 systems containing one or multiple mutations with Isothermal Titration Calorimetry (ITC) determined binding free energy difference upon mutations (Additional file 1: Table S1). In the dataset, 71 of them contain single mutation. A further investigation shows that 85% of the mutations (60 systems) locates within 5 Å of the co-crystallized ligand, which usually exhibits direct interactions with the ligands and may be intuitively thought to lead serious impact on drugs binding. Nevertheless, as shown in Additional file 1: Fig. S1, there is no obvious correlation between the drug-mutation distance (measured by the nearest two atoms located in the ligand and the mutation site) and the binding free energy change of the ligand upon mutations (*r*_p_ = 0.001), implying that the simple observation or traditional experience is not usually valid and more rational investigation should be considered for accurately evaluating the mutation effects on drugs binding. Therefore, MD simulation in conjunction with various protocols of end-point binding free energy calculations was conducted with the purpose to accurately characterize the mutation effects on drugs binding.

### End-point binding free energy calculations based on various simulation protocols

In this section, we systemically investigated the performance of the MM/GBSA and MM/PBSA approaches on the mutated systems, in which the effects of the MD simulation time (including the minimized structures), the dielectric constants and the entropy effects were taken into consideration. Different from the previous studies where the absolute binding free energy was employed for the comparison, here, the comparison was carried out on the binding free energy difference between the mutated and the wild-type systems (∆∆*G* = ∆*G*_MT_ − ∆*G*_WT_). The Pearson correlation coefficient between the predicted binding affinity and the experimental data was used as the metric for various comparisons.

### Performance of the minimized structures

**Fig. 2:**
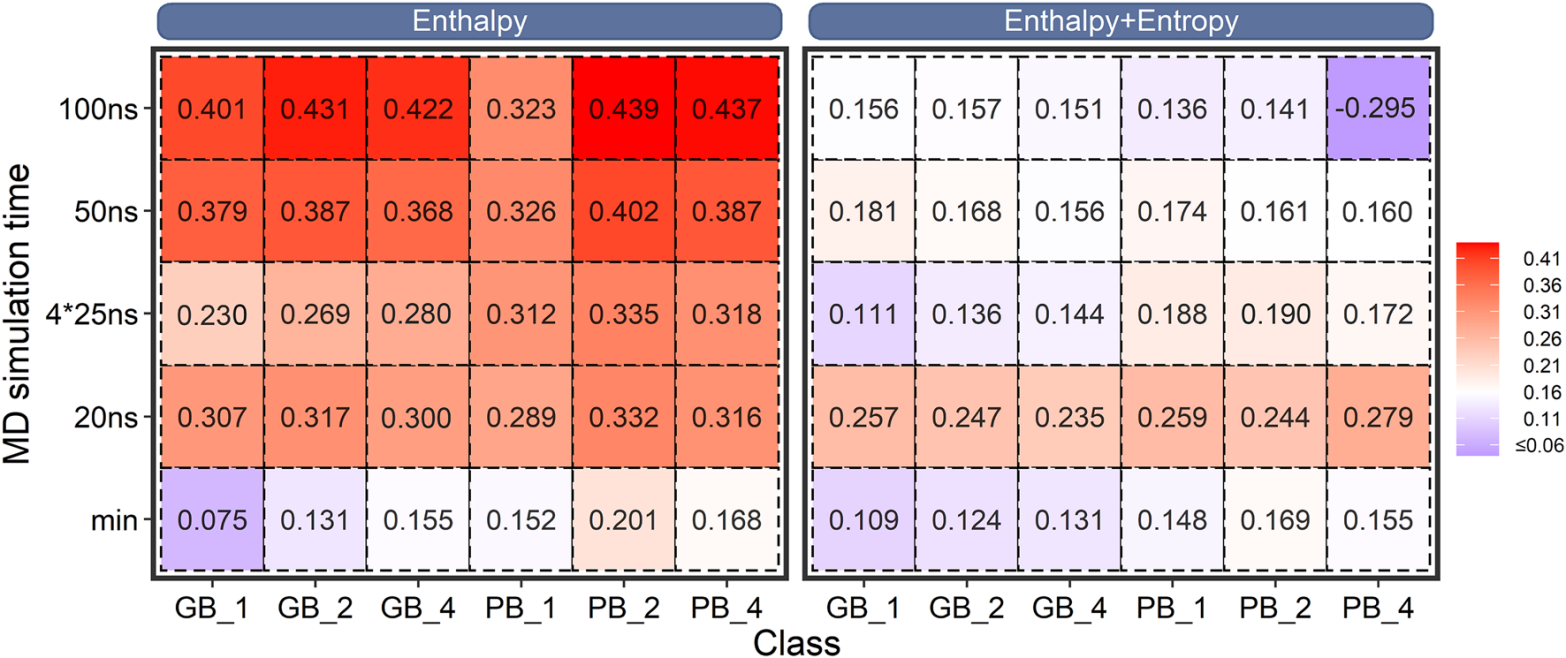
Impacts of MD simulation time, dielectric constant and entropy on the performance of MM/GBSA and MM/PBSA. The Pearson correlation between the predicted binding free energy and the experimental data are colored from blue to red. The left panel shows the accuracy based on the effective binding free energy (enthalpy), while the right panel illustrates the results based on the total binding free energy (enthalpy + entropy). The label *min* corresponds to the result based on the minimized structures. For clear reading, Pearson correlation coefficient less than 0.06 was colored in blue

Our previous work concluded that end-point binding free energy (MM/GBSA and MM/PBSA) estimated based on the minimized structures can give a better predicting accuracy compared with those calculated based on the MD trajectories for the absolute binding free energy ranking [52]. However, it is not clear for the estimation of the relative binding free energy between the mutated and the wild-type systems since the mutated residue may affect the binding free energy of the drug through different orientations or conformations. Thus, herein we performed the MM/GBSA and MM/PBSA calculations with the minimized structures to give a comparison. As shown in the last line of the left panel in Fig. 2, MM/PBSA with the minimized structure using *ε*_in_ = 2 exhibits the best accuracy (*r*_p_ = 0.201), which is much better than the results of MM/GBSA (*r*_p_ = 0.075 ~ 0.155). Nevertheless, even for the best case, the performance of the end-point calculation based on the minimized structures is still worse (*r*_p_ = 0.201), indicating that structural adjustment should be taken into consideration since the manually introduced mutations may impair the microenvironment of the surrounding residues. Encouragingly, significant improvement is shown of the result when carrying out MD simulations for the binding free energy calculations, in which the best *r*_p_s of MM/GBSA and MM/PBSA achieve 0.431 and 0.439, respectively, and are significantly better than the corresponding result based on the minimized structures, implying that MD simulation is a valid approach for the adjustment of the mutation sites to derive a more accurate result.

### Impact of the MD simulation time

As discussed above, a minimized structure may be unable to sample a favorable conformation of the mutated residue to appropriately adjust the binding free energy. Thus, MD simulation is apparently necessary to derive a more reasonable result. Nevertheless, the introduction of MD simulation to end-point binding free energy calculation leads to a new question that how long a MD simulation should be performed to derive a reasonable result. To answer this question, here, different MD simulation time were compared (from 20 to 100 ns or four 25 ns MD trajectories) to give an advisable strategy. As shown in the left panel of Fig. 2, for MM/GBSA at *ε*_in_ = 1, with the extension of the MD simulation time, the Pearson correlation coefficient gradually increases from 0.075 of the minimized structure to 0.401 of the 100 ns MD simulation. A similar tendency is shown of the result based on higher dielectric constants (*ε*_in_ = 2 or 4) of MM/GBSA, where no matter how to adjust the dielectric constant, the accuracy increases with the MD simulation time. Moreover, a similar result is shown of the MM/PBSA result, where the results based on 50 ~ 100 ns MD simulations are much better than those based on the corresponding minimized structures. Nevertheless, comparable best accuracies are shown of MM/GBSA and MM/PBSA at 100 ns MD simulation (0.431 and 0.439 for MM/GBSA and MM/PBSA, respectively, at *ε*_in_ = 2). Moreover, to investigate the performance of MM/GB(PB)SA on the converged part of the MD trajectories, we calculated the standard binding free energy (enthalpy) based on the last 50 ns trajectory for each system. As shown in Additional file 1: Fig. S2, the best accuracy of MM/GBSA and MM/PBSA are 0.428 and 0.421 (*ε*_in_ = 2), respectively, which is comparable with or a bit lower than the corresponding result based on the full 100 ns MD trajectories (0.431 and 0.439 for MM/GBSA and MM/PBSA, respectively, at *ε*_in_ = 2), indicating that the whole conformational ensemble of a MD trajectory contributes to the final energetic change of the ligand upon mutations, and therefore no additional attention needs to be taken to exclude the so-called unconverged samples for binding free energy calculation. Nevertheless, all the production runs (100 ns or the following 4 × 25 ns) were conducted after a heating and an equilibrium stage of MD simulation, which may, to a large degree, mitigate the unfavorable interactions arising from the manually introduced mutations in the initial structures.

**Fig. 3:**
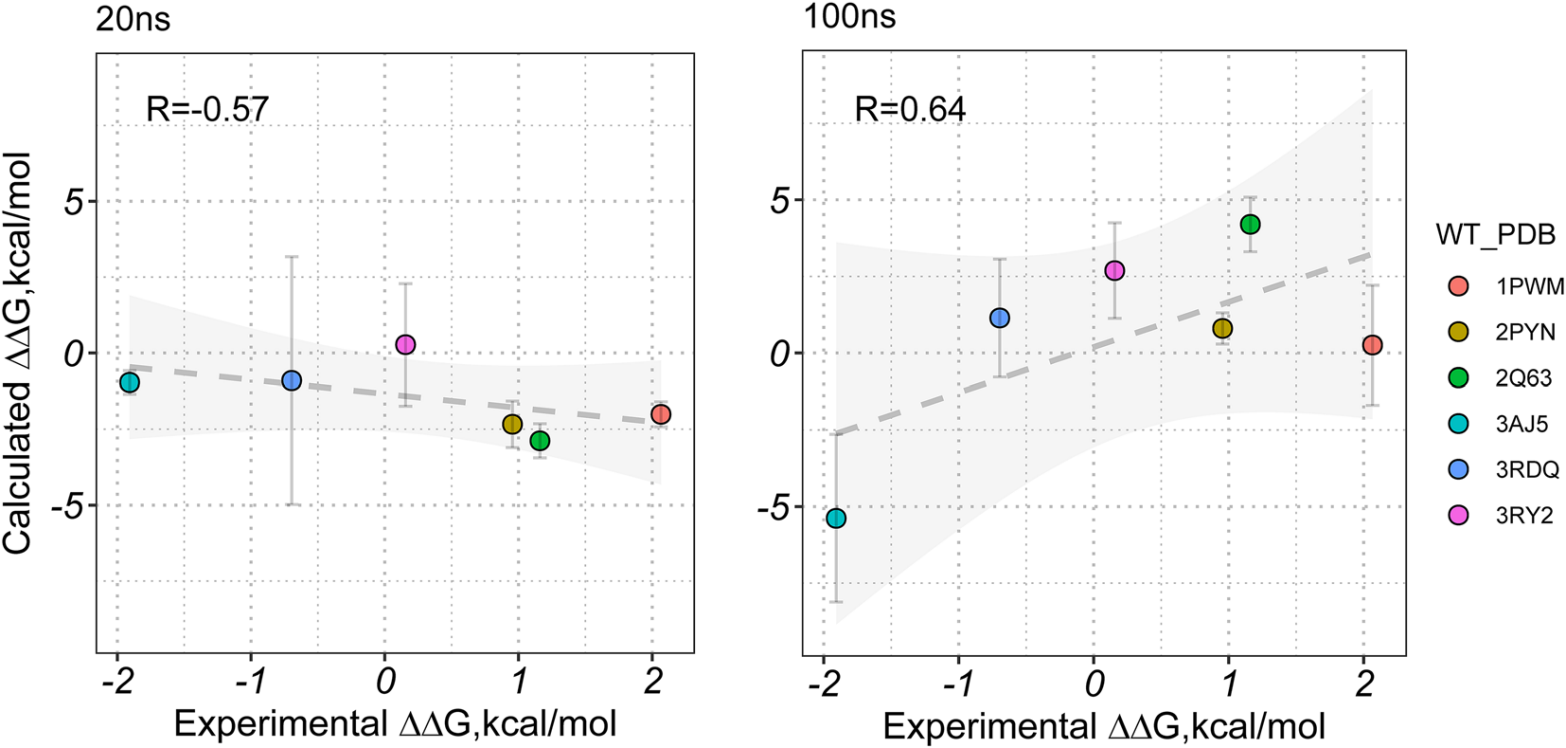
Impact of the MD simulation time on the distant-mutation-containing systems, where the Pearson correlation coefficient can be substantially improved with the extension of the MD simulation time for the MM/GBSA calculation (enthalpy at *ε*_in_ = 2)

Besides, compared with the result based on one single long MD trajectory (100 ns), both MM/GBSA and MM/PBSA exhibit much lower accuracy based on the four short MD trajectories (25 ns for each with the aggregated MD simulation time of 100 ns for each system, the row of “4 × 25 ns” in Fig. 2) with the result reasonably consistent with the first 20 ns result using one single long MD trajectory (namely better than the result based on the minimized structures but worse than the 50 ns MD result), implying that long MD simulation is necessary to be used to adjust the manually introduced mutations to improve the prediction result since the mutations may need long MD time to propagate their effects. Indeed, the analysis of the six distant-mutation-containing systems (with the drug-mutation distance > 8 Å, Additional file 1: Fig. S1) verifies the speculation. As shown in Fig. 3, a short MD simulation (20 ns) may be hard to propagate the mutation effect from a distant mutation to the binding site, thus resulting in a very bad result (*r*_p_ = − 0.57 for MM/GBSA at *ε*_in_ = 2, left panel of Fig. 3), whereas a longer MD simulation (100 ns) is capable of propagating the mutation effect from a distant mutation to the binding pocket, thus substantially improves the prediction result (*r*_p_ = 0.64 for MM/GBSA at *ε*_in_ = 2, right panel of Fig. 3). Moreover, the mechanism of how MD simulation time influences the performance of the predicted binding affinity has been analyzed on a representative system in the last part of this section, which may facilitate one to understand how the mutation effect propagated with the extension of MD simulation time.

### Impact of the dielectric constant

How to choose an appropriate dielectric constant is usually a vital issue in the end-point binding free energy calculations since it may significantly affect the prediction accuracy. Notably, different types of systems may exhibit different tendencies to the employed dielectric constant [38, 50–52, 55–62], such as a higher dielectric constant (i.e. *ε*_in_ = 4) may be a good choice for the protein–ligand systems [51, 52, 63–65], while a medium dielectric constant (i.e. *ε*_in_ = 2) may be more suitable for the protein-peptide [60] and protein-RNA/DNA systems [56], whereas a low dielectric constant (*ε*_in_ = 1) is better for the protein–protein systems [57]. Therefore, to find the suitable dielectric constant for the manually mutated systems, we also used the dielectric constants of 1, 2 and 4 for the MM/GB(PB)SA calculations. As shown in the left panel of Fig. 2, MM/GBSA and MM/PBSA exhibit different preferences: For MM/GBSA, although choosing a medium dielectric constant (*ε*_in_ = 2) may achieve a better result, not much difference is evident from the results based on different dielectric constant (*r*_p_ = 0.401 ~ 0.431 for 100 ns MD result). On the other hand, for MM/PBSA, a relatively higher dielectric constant (*ε*_in_ = 2 and 4) can generate a better accuracy (*r*_p_ = 0.439 under the 100 ns MD simulations with *ε*_in_ = 2). Nevertheless, comparable accuracies are demonstrated for the standard MM/GB(PB)SA calculations (without considering the entropy, left panel in Fig. 2) when using any dielectric constant (*ε*_in_ = 1 ~ 4) for MM/GBSA calculations and a relatively higher dielectric constant (*ε*_in_ = 2 and 4) for MM/PBSA calculations. This result indicates that, different from the absolute binding free energy calculation, where the predicted binding free energy may be significantly affected by the use of different dielectric constants [51], the relative binding free energy calculation between e.g. the mutants and the wild-type system (∆∆*G*) may largely cancel out the difference of the electrostatic effects at different dielectric constants, making the systems insensitive to the dielectric constant.

### Impact of the conformational entropy

Another important issue for the end-point binding free energy calculation is whether it is necessary to incorporate the entropy term for the standard MM/GB(PB)SA calculations (the so called effective binding free energy or enthalpy) since the incorporation of entropy may not be able to improve the correlation between the predicted binding free energies and the experimental data in most cases [50]. However, different voices arise in various system-specific studies, where incorporating entropy into the standard MM/GB(PB)SA calculations helps not only to reveal the binding mechanisms, such as drug resistance [23], but also to reproduce the absolute binding free energy against the experimental data. Therefore, here the entropy effect has also been taken into consideration for the accuracy investigation. The structure-truncation strategy was employed for the entropy calculation since the too high computational cost of the normal mode entropy (NME) calculation. As shown in the right panel of Fig. 2, unfortunately, incorporating entropy into the standard MM/GB(PB)SA calculations seriously impairs the prediction accuracy, where no obvious rules can be summarized from the result. Nevertheless, since the characterization of the mutation effect usually involves the relative binding free energy calculation (∆∆*G* between the mutated and the wild-type systems), where the entropy contributions can be largely canceled out between systems, there is no necessary to incorporate the entropy term into the predicted binding free energies.

### Impact of the mutation properties and target specificity on the predicting accuracy

**Fig. 4:**
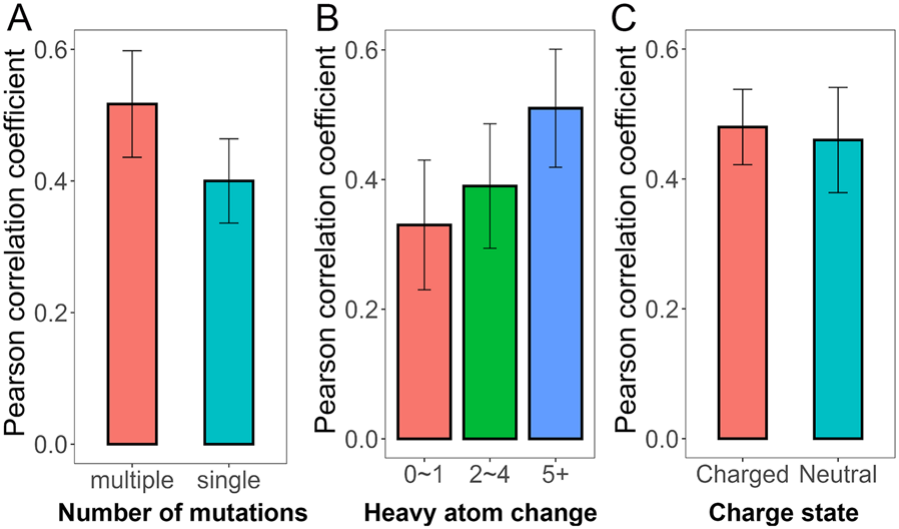
Impact of mutation properties on the predicting accuracy. **A** Single or multiple mutation(s), **B** the number of heavy atoms change in mutations, and **C** the change of charge state of the mutation on the predicting accuracy. All the investigation were performed based on MM/GBSA at *ε*_in_ = 2 and 100 ns MD simulation

To further investigate the impacting factors on the performance of the predicted binding free energies upon mutations, the difference of the mutated and the original residues is analyzed based on MM/GBSA under the condition of *ε*_in_ = 2 and 100 ns MD simulation. As shown in Fig. 4, here, three main impacting factors were investigated, including the number of mutations in the targets (single or multiple mutation(s), Fig. 4A), the number of heavy atoms change between the mutated and the original residues (Fig. 4B), and the change of charge state of the mutation (Fig. 4C). The distribution of each property on the investigated targets can be found in Additional file 1: Figs. S3–S5.

For the investigation of the number of mutations in a system (Fig. 4A), it shows that systems involving multiple mutations exhibit higher predicting accuracy (*r*_p_ = 0.517) compared with those involving only one mutation (*r*_p_ = 0.400). It is easy to understand that systems involving multiple mutations may generate larger impact on the binding of a ligand to its receptor compared with the original system, and thus can be more accurately predicted. Moreover, the further investigation on the number of heavy atoms change between the mutated and the original systems also shows a same tendency (Fig. 4B), where the predicting accuracy improves with the increased change of the heavy atom count between the mutated and the original residues (with the *r*_p_ of 0.335 for 0 ~ 1 heavy atom change, 0.393 for 2 ~ 4 heavy atom change, and 0.511 for > 5 heavy atom change), implying that the impact of large change between the mutated and the original residues is much easier to be predicted (e.g. systems with multiple mutations or with > 5 heavy atoms change) than those with tiny difference between the mutated and the wild-type residues (e.g. systems with single mutation or with 0 ~ 1 heavy atom change). Besides, the impact of charge-state change of the mutations is also investigated, where if one residue (before or after mutation) involves charged residue (ASP, GLU, LYS and ARG), the system is assigned to the charged group, otherwise, it will be assigned to the neutral group. As shown in Fig. 4C, comparable result is shown of the neutral (*r*_p_ = 0.458) and the changed groups (*r*_p_ = 0.478), suggesting that the change of charge state of the mutated residue(s) may not affect the accuracy of the predictions so much because of the well parameterized protein force field.

**Fig. 5:**
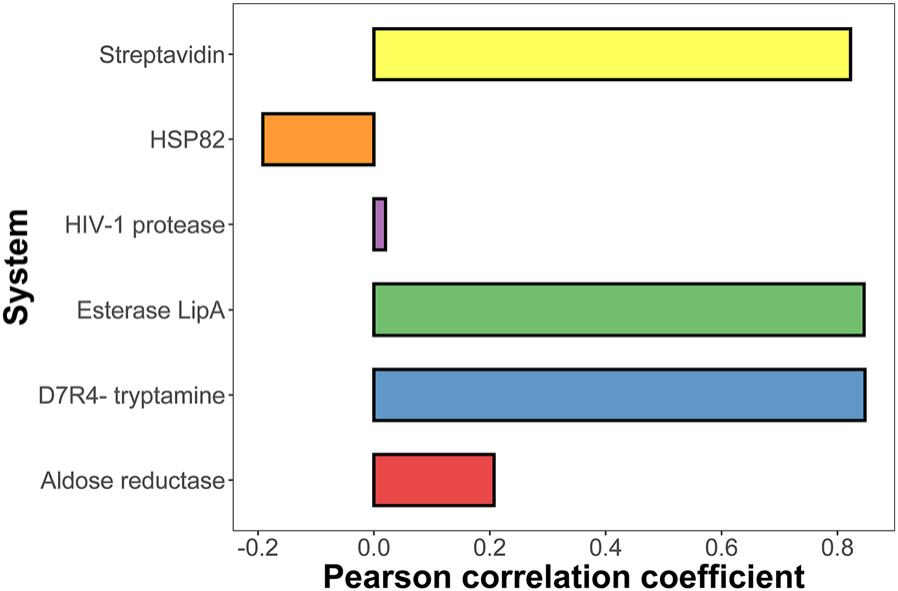
Performance of MM/GBSA on specific targets based on *ε*_in_ = 2 and 100 ns MD simulation

Furthermore, we also investigated the system dependency of MM/GBSA on characterizing the mutation effect for specific proteins, where systems with ≥ 6 individuals were plotted based on *ε*_in_ = 2 and 100 ns MD simulation. As shown in Fig. 5, the Pearson correlation coefficient is higher than 0.8 in the systems of Esterase-LipA, Steptavidin and D7R4-tryptamine, whereas lower than or around 0 in the systems of HSP82 and HIV-1 protease. Although one may concern that too few samples were incorporated in each protein group, the predicted result of some systems (e.g. HIV-1 protease) is consistent with the existing result reported by previous studies on the same system, such as the previous investigation on 220 HIV-1 protease systems shows the best Pearson correlation coefficient of 0.165 for the absolute binding free energy calculation using MM/GBSA [51], which is consistent with the current observed low accuracy of the HIV-1 protease group on the relative binding free energy calculation. The deeper reason may attribute to complicated chemical structures of the HIV1-protease inhibitors (usually containing > 100 atoms and > 10 rotatable bonds), and thus is hard to be accurately predicted by the classical force field. Therefore, we emphasize that caution should always be minded when using a computational method.

### Insights into the impact of MD simulation time on the mutated system

As shown in the preceding section, the length of MD simulation time is of crucial importance in regulating the performance of the end-point binding free energy in the mutated systems, where it can be concluded that longer MD simulation (e.g. 100 ns) benefits the relative binding free energy calculation (∆∆*G*) for both MM/GBSA and MM/PBSA. To better understand the role of MD simulation time on the mutated systems, here a case analysis is performed on the system of D7R4-tryptamine (including 10 mutants).

**Fig. 6:**
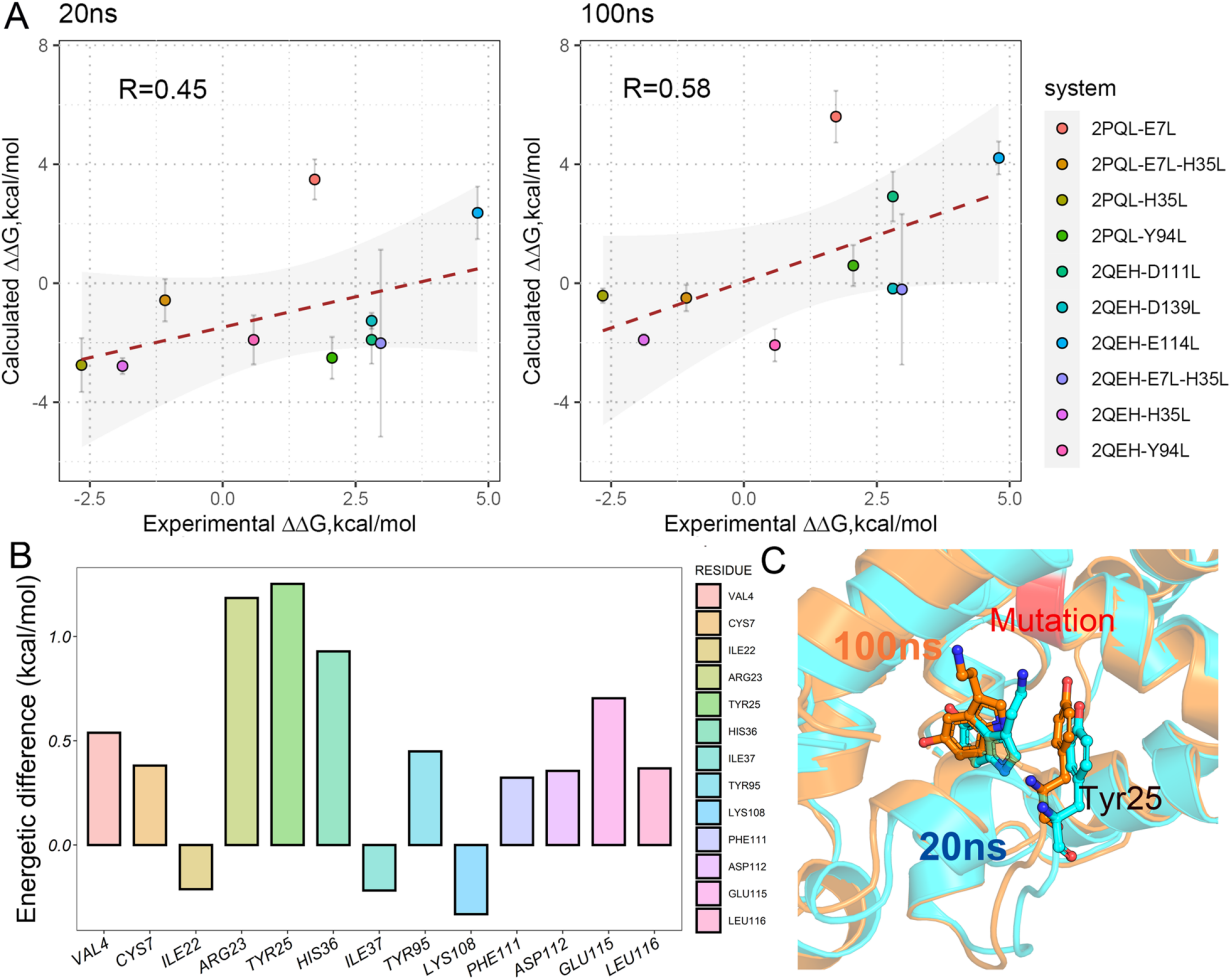
Correlation between the predicted binding free energy difference and the experimental data of the D7R4-tryptamine systems in different MD simulation time, where the standard MM/GBSA results based on 20 and 100 ns MD simulation (*ε*_in_ = 1) are shown in panel A. The energetic difference of the vital residues between the D111L mutant and the wild-type system is shown in panel B with the corresponding most populated conformations illustrated in panel C, in which structures derived from 20 and 100 ns MD simulations are colored in blue and orange, respectively, with the mutation site colored in red

As shown in Fig. 6A, the Pearson correlation coefficient of the standard binding free energy predicted by MM/GBSA (enthalpy at *ε*_in_ = 1) improves with the extension of MD simulation time from 0.45 to 0.58 in the 20 ns and 100 ns results, respectively, where 5 systems in 10 show correct tendency (consistent sign with the experimental binding free energy difference) of the predicted relative binding free energy against the experimental data in 20 ns MD simulation, whereas 7 out of the 10 systems exhibit correct result in the 100 ns MD simulation. In the two additionally correctly adjusted systems (D111L and Y94L mutants, Fig. 6A), the relative binding free energy of the D111L mutant changes remarkably (with > 4 kcal/mol binding free energy difference between the 100 ns and the 20 ns results), thus we investigated this mutant to reveal how the extension of the MD simulation time ameliorates the result.

As shown in Table 1, since the relative binding free energy calculation incorporates two systems (namely, the wild-type and the mutated systems), we illustrated the MM/GBSA binding free energy for both systems in each reference time point (20, 50 and 100 ns) to give a comparison. It can be found that the calculated binding free energy of the wild-type system is very stable across the whole 100 ns MD simulation with the energetic difference between the 20 and 100 ns results < 1 kcal/mol (∆∆*G*_WT_100ns-20ns_ = − 0.56 kcal/mol), whereas large energetic difference is shown of the D111L-mutated system (∆∆*G*_MT_100ns-20ns_ = 4.25 kcal/mol). The reason why so large energetic difference happens in the mutated system may be attributed to the manually introduced mutation that may probably perturb the surrounding residues of the protein. Therefore, we further decomposed the total binding free energy into residue level to reveal a more detailed picture. Figure 6B illustrates the most energetic difference contributed residues, where TYR25 exhibits the most significant energetic change between the mutated and the wide-type systems. Further superimposition of the most populated conformations of the wild-type and the mutated systems shows that, although the conformation of TYR25 does not change dramatically during the 100 ns MD simulation, the sidechain of the ligand exhibits an obvious conformational shift (Fig. 6C) that does not occur at the beginning of MD simulation, but gradually shifts with the extension of the MD simulation and finally results in remarkably attenuated interaction to TYR25 (− 3.39 *versus* − 2.24 kcal/mol in the 20 and 100 ns MD results, respectively), implying that long time MD simulation is capable of re-adjusting the distribution of the micro-environment of the protein around the mutation site, thus leading to more reasonable energetic result against the experimental data.

**Table 1:** Energetic difference of the D7R4-tryptamine systems in the 20, 50 and 100 ns MD simulations based on MM/GBSA (kcal/mol, *ε*_in_ = 1)

	20 ns	50 ns	100 ns	∆∆*G*_100ns-20ns_
WT	− 20.95	− 21.57	− 21.51	− 0.56
D111L	− 22.85	− 19.78	− 18.60	4.25

## Conclusion

In this study, comprehensive analyses were performed to investigate the mutation effects on the performance of end-point binding free energy calculations. Compared with the alchemical methods, end-point binding free energy calculations represented by MM/GBSA and MM/PBSA are much computationally cheaper with reasonable accuracy (with the best *r*_p_ ~ 0.44 on a challenging dataset), thus are useful for application in large-scale mutation associated studies.

Specifically, the current result shows that a relatively long MD simulation (e.g. 100 ns) usually benefits the prediction accuracy in both MM/GBSA and MM/PBSA, in which MM/GBSA is insensitive to the dielectric constant, while MM/PBSA prefers a relatively higher dielectric constant (*ε*_in_ = 2 or 4). Overall, MM/GBSA and MM/PBSA give a comparable best accuracy in the MD-based calculations (with the Pearson correlation coefficients of 0.431 and 0.439 for MM/GBSA and MM/PBSA, respectively), while MM/PBSA performs remarkably better than MM/GBSA in the minimized structures though the best Pearson correlation coefficient is very low (*r*_p_ = 0.201).

Moreover, analyses of the mutation properties to the prediction accuracy show that systems suffering from large perturbations (e.g. multiple mutations or large number of heavy atoms change in the mutation site) are much easier to be accurately predicted due to the significant change of the systems. Besides, a system of D7R4-tryptamine was employed as an example to reveal the impact of MD simulation time on the mutation effect, where conformational change of the ligand caused by the manually introduced mutation is found responsible for the adjustment of the binding free energy difference along the MD trajectory, indicating that manually modeled structures should be well adjusted by strategies such as MD simulation to match the micro-environment of the protein.

## Supplementary information

**Additional file 1.** Additional Tables and Figures.

## Data Availability

All the initial structures, resulting data and scripts for AMBER simulations (including the four stages of minimization, three stages of MD simulation and MM/PB(GB)SA/NMA calculation) were made available in GitHub (https://github.com/yuyangniuer/MUTATION).
