# Supplementary material for: Predicting the mutation effects of protein–ligand interactions via end-point binding free energy calculations: strategies and analyses
Source: J Cheminform. 2022 Aug 20;14:56. doi: 10.1186/s13321-022-00639-y (PMC9392442; doi:10.1186/s13321-022-00639-y)
Supplement: Supplementary file 1 — Additional file 1. Additional Tables and Figures. [file 13321_2022_639_MOESM1_ESM.pdf]

Supporting Information

Predicting the Mutation Effects of Protein-Ligand Interactions via End-Point Binding Free Energy Calculations: Strategies and Analyses

Yang Yu[1,#], Zhe Wang[2,#], Lingling Wang[1], Sheng Tian[3], Tingjun Hou[2,*] and Huiyong Sun[1,*]

[1]Department of Medicinal Chemistry, China Pharmaceutical University, Nanjing 210009, Jiangsu, P. R. China.

[2]Innovation Institute for Artificial Intelligence in Medicine of Zhejiang University, College of Pharmaceutical Sciences, Zhejiang University, Hangzhou 310058, Zhejiang, P. R. China.

[3]Department of Medicinal Chemistry, College of Pharmaceutical Sciences, Soochow University, Suzhou 215123, P. R. China

[#]These authors contributed equally to this work.

Corresponding authors:

Tingjun Hou

E-mail: tingjunhou@zju.edu.cn

Huiyong Sun

Email: huiyongsun@cpu.edu.cn

Table S1. Basic information of the investigated systems.

Protein Name	PDB ID	Distance (Å)[a]	Mutation Site	$\Delta\Delta G_{exp}$ (kcal/mol)[b]	Ligand Name
Aldose reductase	2IKI	2.93	T113**Y**	2.07	388
	2IKI	3.95	V47**I**	-0.31	388
	3LEP	2.93	**T**113C	0.14	388
	3LQG	2.93	**T**113A	0.77	388
	3M4H	2.93	**T**113V	1.54	388
	2PDJ	2.28	**L**300A	0.46	393
	2PDP	6.87	**S**302R	2.52	393
	2PDU	4.13	**C**303D	2.47	393
	2PZN	6.87<br>4.13	S302**R**<br>C303**D**	2.47	393
	2PZN	2.79	T113**Y**	2.33	393
	2PDG	4.30	L300**A**	-0.24	47D
	2PDG	7.16<br>4.18	S302**R**<br>C303**D**	2.74	47D
	2PDG	3.50	T113**Y**	2.59	47D
	2PDG	4.72	V47I	1.20	47D
	2PDN	7.16	**S**302R	2.79	47D
	2PDQ	4.18	**C**303D	2.40	47D
	1PWM	2.95	L300**A**	0.46	FID
	1PWM	3.71	L301**M**	0.70	FID
	1PWM	9.44	T113**Y**	2.07	FID
	1PWM	4.18	C303**D**	1.03	FID
	1PWM	4.80	F121**P**	0.29	FID
	1PWM	4.30	L300**P**	1.87	FID
	1PWM	7.16<br>4.18	S302**R**<br>C303**D**	1.90	FID
	1PWM	3.96	V47**I**	0.14	FID
	2PD9	3.96	**V**47I	0.14	FID
	2PDW	7.43	**C**303D	1.03	FID
	2PDY	7.16<br>4.18	**S**302R<br>**C**303D	1.90	FID
	1US0	2.85	T113**C**	-1.52	LDT
	3LD5	2.85	**T**113C	1.00	LDT
	3LQL	2.85	**T**113A	1.20	LDT
	3LZ5	2.85	**T**113V	0.77	LDT
Anti-tumor lectin	3AFK	4.27	E66**A**	-0.62	TFA
	3AFK	3.38	R85**A**	1.12	TFA
	3M3C	4.27	E66**A**	-0.49	TFA
	3M3C	3.38	R85**A**	0.63	TFA
HSP82	2VWC	6.09<br>3.84<br>4.29	L89**V**<br>L93**I**<br>V136**M**	0.51	BC2
	2VWC	4.29	V136**M**	0.40	GDM
	1A4H	3.59	E88**G**	-0.11	GDM
	1A4H	2.72	K44**R**	0.24	GDM
	1A4H	2.72<br>4.00	K44**R**<br>K98**N**	0.66	GDM
	1A4H	4.00	K98**N**	-0.36	GDM
	1A4H	6.26	L89**V**	-0.95	GDM
	1A4H	6.26<br>3.51	L89**V**<br>L93**I**	-0.52	GDM
	1A4H	3.51	L93**I**	-0.74	GDM
	1A4H	3.47	N92**L**	0.19	GDM
	1A4H	4.43	V136**M**	0.49	GDM
	1A4H	6.26<br>3.51<br>4.43	L89**V**<br>L93**I**<br>V136**M**	0.32	GDM
	2YGE	3.59<br>3.40	E88**G**<br>N92**L**	-1.26	GDM
	2YGF	6.26<br>3.51	**L**89V<br>**L**93I	0.32	GDM

		4.43	**V**136M		
RolR	3AQT	5.01	D94**A**	1.76	RCO
	3AQT	3.58	R145**A**	2.11	RCO
	3AQT	3.65	R148**A**	1.45	RCO
Esterase LipA	3H2K	3.22	G221**I**	0.26	BOG
	3H2K	3.04	G231**A**	1.21	BOG
	3H2K	3.04	G231**F**	1.92	BOG
	3H2K	3.04	G231**I**	1.37	BOG
	3H2K	2.96	N228**W**	0.23	BOG
	3H2K	3.80	S176**A**	0.05	BOG
HIV-1 protease	2PYM	7.05	**N**88D	1.52	1UN
	2PYN	16.04	**A**71V	0.95	1UN
	2Q63	9.57	**L**90M	1.16	1UN
	2Q64	16.04<br>2.33	**A**71V<br>**D**30N	-1.15	1UN
	2Q64	2.33	**D**30N	1.78	1UN
	2Q64	9.57<br>2.33	**L**90M<br>**D**30N	-0.47	1UN
	2Q64	7.05<br>2.33	**N**88D<br>**D**30N	-1.15	1UN
	2FGU	3.83	**T**80**V**	0.47	ROC
	2FGU	3.83	**T**80S	0.16	ROC
PMT	3UJB	3.68	H132**A**	0.26	SAH
	3UJB	3.52	Y19**F**	0.25	SAH
Streptavidin	1DF8	3.75	**S**45A	4.06	BTN
	3RDO	3.53<br>3.17<br>1.40<br>8.20<br>9.08<br>10.81	**T**90S<br>**W**108V<br>**L**110T<br>**F**29L<br>**S**52G<br>**R**53S	4.62	BTN
	3RY2	9.08	S52**G**	0.16	BTN
	3RY2	9.08<br>10.81	S52**G**<br>R53**D**	0.31	BTN
	3RDQ	9.85	**S**52G	-0.70	DTB
	3RDQ	3.92<br>3.21<br>2.97<br>7.90<br>9.85<br>11.14	**T**90S<br>**W**108V<br>**L**110T<br>**F**29L<br>**S**52G<br>**R**53S	-0.30	DTB
Glutathione Transferase	1K3L	4.95	I219**A**	1.73	GTX
Major Urinary Protein	1QY1	2.54	Y120**F**	1.12	PRZ
D7R4-<br>tryptamine	2PQL	3.93	E7**L**	1.73	TSS
	2PQL	3.93<br>3.57	E7**L**<br>H35**L**	-1.09	TSS
	2PQL	3.57	H35**L**	-2.66	TSS
	2PQL	2.97	Y94**L**	2.05	TSS
	2QEH	2.81	D111**L**	2.80	Serotonin
	2QEH	4.75	D139**L**	2.80	Serotonin
	2QEH	2.73	E114**L**	4.80	Serotonin
	2QEH	3.93<br>3.57	E7**L**<br>H35**L**	2.97	Serotonin
	2QEH	3.57	H35**L**	-1.89	Serotonin
	2QEH	2.97	Y94**L**	0.58	Serotonin
Main hemagglutinin component	3AJ5	18.01	W176**A**	-1.91	NGA
Beta-D-Galactose	3AH4	2.2	F179**I**	0.221	GAL

[a]The drug-mutation distance was measured based on the nearest two atoms coming from the ligand and mutation site of the protein. [b]The difference of the binding free energy between the wild-type and the mutants was calculated by $\Delta\Delta G=\Delta G_{\mathrm{MT}}-\Delta G_{\mathrm{WT}}$. The manually mutated residues were marked in bold.

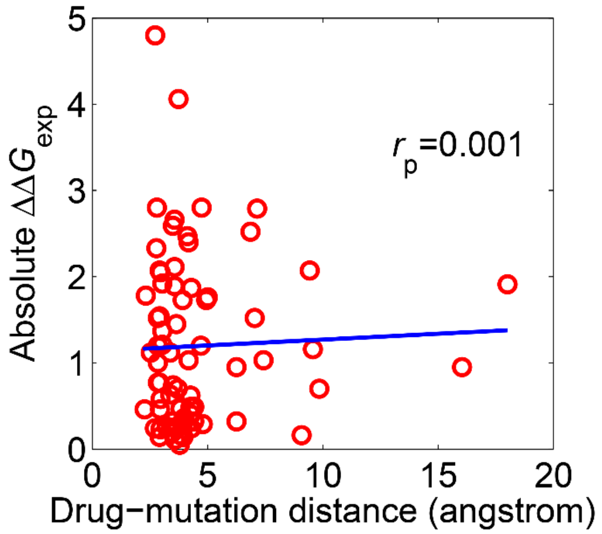

Figure S1. Correlation between the drug-mutation distance and the absolute binding free energy change upon mutations, where the drug-mutation distance was measured based on the nearest two atoms coming from the ligand and mutation site of the protein.

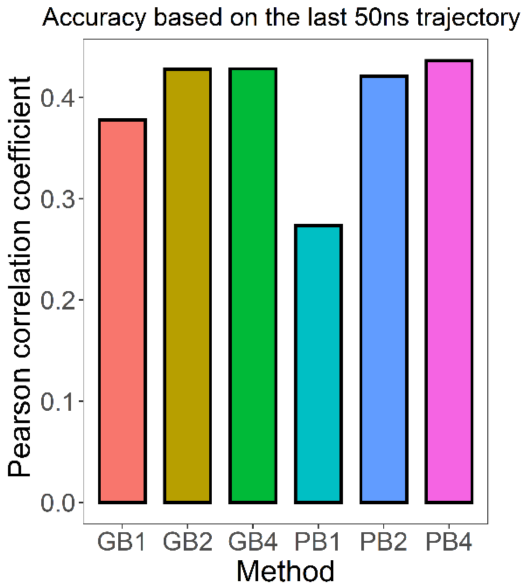

Figure S2. Pearson correlation coefficients based on the last 50 ns MD trajectories for the enthalpies predicted under different dielectric constants of MM/GBSA and MM/PBSA.

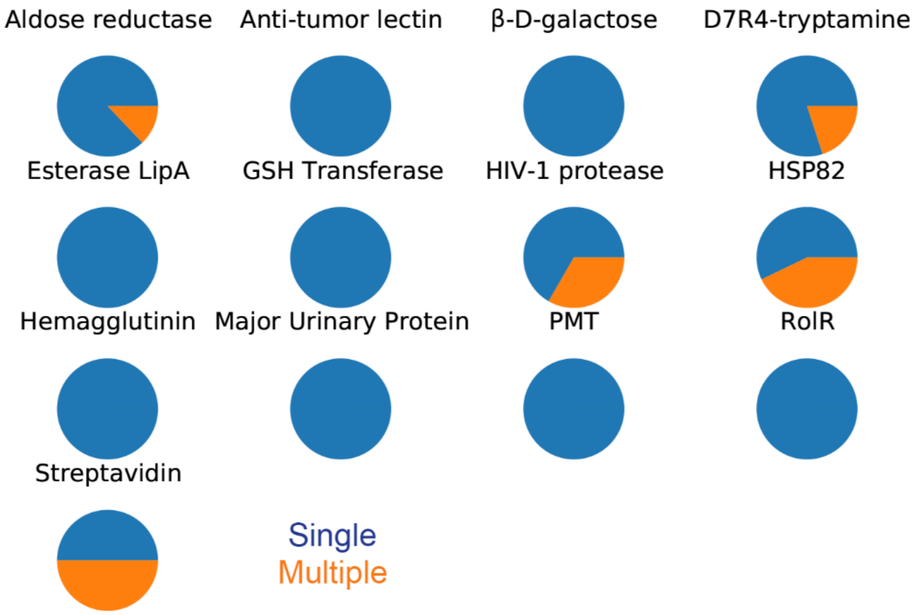

Figure S3. Distribution of the number of mutations in the systems (**single:** 71 systems; **multiple:** 18 systems)

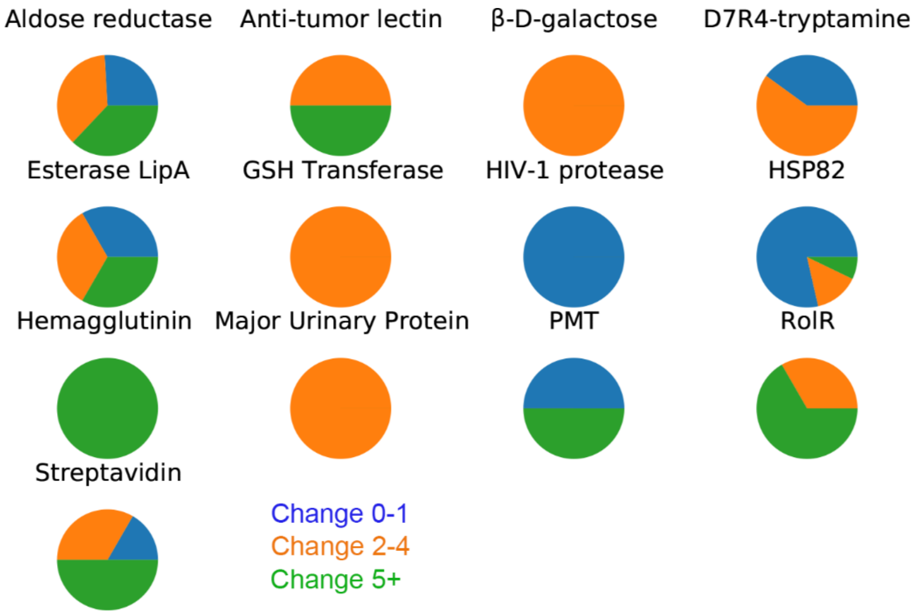

Figure S4. Distribution of the heavy atoms change of the systems (**0~1:** 37 systems; **2~4:** 30 systems; and **≥5:** 22 systems)

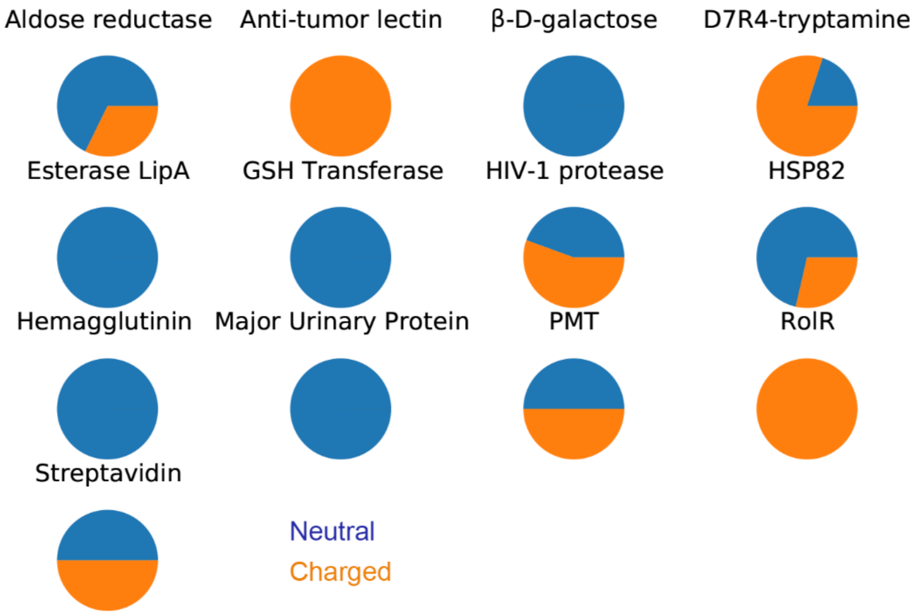

Figure S5. Distribution of the charge state change of the systems (**charged:** 38 systems; and **neutral:** 51 systems)
